# Investigation of Photoplethysmography Behind the Ear for Pulse Oximetry in Hypoxic Conditions with a Novel Device (SPYDR)

**DOI:** 10.3390/bios10040034

**Published:** 2020-04-04

**Authors:** Brian Bradke, Bradford Everman

**Affiliations:** 1 Department of Mechanical Engineering, The David Crawford School of Engineering, Norwich University, 158 Harmon Drive, Northfield, VT 05663, USA; 2 Spotlight Labs, 13335 15 Mile Rd Ste-135, Sterling Heights, MI 48312, USA; everman@spotlightlabs.com

**Keywords:** pulse oximetry, oxygen saturation, pulse rate (PR), physiological monitoring, SPYDR, vital signs monitor, photoplethysmography (PPG), noninvasive monitoring, hypoxia

## Abstract

Photoplethysmography (PPG) is a valuable technique for noninvasively evaluating physiological parameters. However, traditional PPG devices have significant limitations in high-motion and low-perfusion environments. To overcome these limitations, we investigated the accuracy of a clinically novel PPG site using SPYDR^®^, a new PPG sensor suite, against arterial blood gas (ABG) measurements as well as other commercial PPG sensors at the finger and forehead in hypoxic environments. SPYDR utilizes a reflectance PPG sensor applied behind the ear, between the pinna and the hairline, on the mastoid process, above the sternocleidomastoid muscle, near the posterior auricular artery in a self-contained ear cup system. ABG revealed accuracy of SPYDR with a root mean square error of 2.61% at a 70–100% range, meeting FDA requirements for PPG sensor accuracy. Subjects were also instrumented with SPYDR, as well as finger and forehead PPG sensors, and pulse rate (PR) and oxygen saturation (SpO_2_) were measured and compared at various reduced oxygen profiles with a reduced oxygen breathing device (ROBD). SPYDR was shown to be as accurate as other sensors in reduced oxygen environments with a Pearson’s correlation >93% for PR and SpO_2_. In addition, SPYDR responded to changes in SpO_2_ up to 50 s faster than PPG measurements at the finger and forehead.

## 1. Introduction

The measurement of physiological metrics, such as pulse rate and oxygen saturation, is of great interest to research, clinical, and commercial communities. Recent technological advancements have augmented our ability to access and evaluate physiological parameters related to human health and performance unlike ever before [1]. Technologies for accurately monitoring pulse rate (PR) and oxygen saturation (SpO_2_) are especially sought after for numerous applications, such as the ability to evaluate general health, and changes in physical and cognitive performance [2,3]. One of the most common techniques for evaluating measurements of blood oxygen saturation and cardiovascular performance is pulse oximetry by photoplethysmography (PPG) [4,5,6]. Photoplethysmography is a simple, non-invasive, low cost, and portable optical technique that can measure pulse rate, oxygen saturation, blood pressure, and cardiac output, and it is generally accepted as providing valuable information about the general functioning, as well as acute changes, of the cardiovascular system [2,7].

A typical PPG device utilizes a light source and photodetector to measure changes within subcutaneous microvasculature to measure physiological data on the cardiovascular system from sites on the skin’s surface. The differential absorption of two wavelengths in pulsatile blood flow by oxygenated and deoxygenated hemoglobin allows for an accurate estimation of arterial oxygen saturation [8,9]. There are two general types of PPG techniques commonly used today, transmission and reflectance. Transmission PPG sensors detect light passed through the tissue and are therefore commonly used on peripheral sites, most commonly fingers and earlobes. Reflectance PPG sensors utilize a light detector adjacent to the emitter and are ideal for single-point contact readings [10]. The most widely utilized locations for reflectance PPG includes the wrist, forearm, ankle, forehead, and torso [11,12]. Typically, these sensors are positioned on the skin using cuffs, or clips, as there is a required amount of pressure needed to apply the sensor to obtain the most accurate and reliable signal [4,13].

While PPG has been studied and used at various anatomical locations with varied applications, there are a number of limitations to the accuracy and effectiveness of these techniques, especially in dynamic or hypoxic environments [6,14]. It is known that the location of the sensor, high motion artifact, and issues with implementation of skin contact may compromise the effectiveness of the PPG sensor measurements [4,12,15]. Tissue alterations, caused by both voluntary and involuntary movements, such as muscle contraction or dilation of tissues, can disrupt sensor readings, as physical displacement of the sensor from its original location can modify the PPG signal by changing the path of light [16,17]. Other factors influencing PPG reading accuracy include other anatomical or physiological differences such as skin color and amount of fluid retained by the tissues [15,18,19].

Because of these various limitations of different PPG sensors at various anatomical locations, many studies have been conducted exploring their accuracy under different conditions. Other studies have collectively compared PPG at the finger, forearm, earlobe, ear canal, wrist, shoulder, forehead, chest, temple, neck, rib cage, wrist, lower back, and tibia [12,20,21,22,23,24,25]. The consensus was that the forehead and finger locations provide the most accurate PR and SpO_2_ measurements in static conditions, although both were found to be susceptible to deleterious effects from varying dynamic conditions, especially movement and desaturation events. While in a number of cases, the finger location was found to produce the most accurate results, Pertzov et al. determined that a centrally located PPG sensor, at the earlobe in their case, is more accurate than at the extremities like the finger and allows for earlier identification, treatment, and resolution of desaturation events [26]. PPG sensor readings at the finger were found to be especially disrupted by movement. A study by Longmore et al. revealed that, among many anatomical locations tested, the forehead was the only location to record heart rate and SpO_2_ within acceptable values both at rest and while walking, while the finger location readings were highly disrupted by movements [12]. Conversely, in another study by Ross et al. comparing PPG sensors at high altitudes at the finger, forehead, and ear lobe, gold standard arterial blood gas results revealed the finger location to perform best, followed by the earlobe location and finally the forehead with the lowest performance for detecting hypoxia [23]. Studies investigating the in-ear location for PPG sensors have found this location to be sufficiently accurate for detecting heart rate and oxygen saturation levels relative to the finger location and advantageous as a centrally located PPG sensor site [21,25,27]. However, a study by Passler et al. determined that motion artifact due to normal jaw movements from chewing gum and talking throughout data recording had a significant deleterious influence on the in-ear location, resulting in a signal interference too intense to determine a precise pattern of pulse rate [28].

While the finger and forehead sensor locations were found to be the most reliable and are the standard for validation of novel sensors, we can see they have some limitations. PPG readings at peripheral sites, like the fingers, are especially negatively influenced by motion artifacts, and other physiological factors, including blood pooling, blood pressure, and altered physiological responses [29,30]. PPG signals from the forehead are less affected by vasoconstriction and blood pooling compared to fingers [31,32]. However, there is a strong venous component to the forehead, as well as larger size of arteries and veins, which deteriorates the quality of the signal and increases unreliability of the measurements [33]. Problems with motion artifact also negatively impact the accuracy and reliability of PPG readings at the forehead, as muscles in the forehead can create regular movement of the sensor over the skin, disrupting measurements [34,35].

Based on these known limitations of current PPG sensor locations, we determined that conditions of ideal PPG sites are ease of accessibility, good microvasculature, without large veins or arteries nearby, no hair or follicles, and low potential for motion artifact, in part affected by musculature, but also by location on the body. For example, the head typically moves less than the extremities and, as such, is a more ideal location for accuracy and reliability of readings in most dynamic circumstances. The location should also be centrally located and most directly in line with the heart to the brain to detect critical desaturation events as quickly as possible.

**Figure 1:**
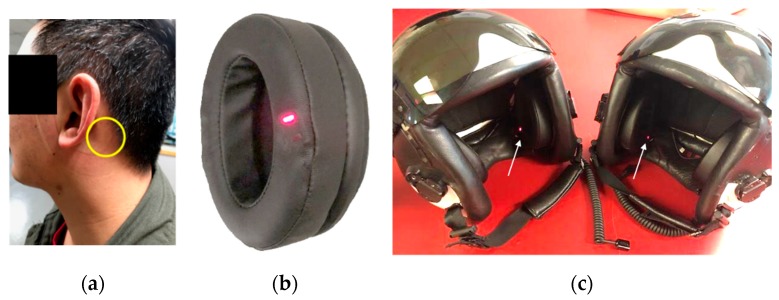
(**a**) Photograph of a test subject after removing helmet instrumented with SPYDR earcups. The yellow circle indicates optimal anatomical placement of the SPYDR sensor when worn. (**b**) Photograph of the SPYDR ear-cup. The multi-wavelength plethysmography sensor is seen as the red light embedded in the ear-seal. (**c**) Photograph of Navy flight helmets HGU-68, configured for tests with SPYDR installed. The white arrow indicates the red light of the sensor.

To meet these criteria and overcome the challenges of capturing accurate and reliable measurements, especially in critical or dynamic environments, we selected a largely unexplored location behind the ear, on the mastoid process, between the pinna and the hairline, above the sternocleidomastoid muscle, and near the posterior auricular artery for our SPYDR^®^ sensor suite (Figure 1a). This mounted behind the ear location is most directly in line from the heart to the brain, is easily accessible, has little or no subcutaneous musculature or ligaments, is highly vascularized with no large arteries or veins, and is not in the extremities, subjected to higher motion and other location-based physiological alterations. Although, this location was previously demonstrated to be ideal mechanically and physiologically by He et al., it still remains largely unexplored and underutilized as a site for a reliable wearable vital signs monitor [36]. The sensor in their study was found to experience deleterious effects of motion artifacts, but we believe this is related to the design on the sensor, not the location.

In order to further optimize PPG measurements at this location behind the ear, we developed an earcup-mounted device with a reflectance PPG sensor embedded within the ear-seal shown in Figure 1b,c, called SPYDR^®^ (Standalone Performance Yielding Deliberate Risk). SPYDR employs an independently manufactured medical grade FDA-approved reflectance PPG sensor (OEMIII with 8000R reflectance sensor, NONIN^®^, Plymouth, MN, USA) that is multi-wavelength (660 nm and 980 nm as pre-calibrated by the manufacturer) within the ear-seal of ear-muff type hearing protection devices like those found in aviation headsets and helmets (Figure 1c). While embedded in the ear-seal, the sensor is up against the skin, with nothing else between the sensor and the skin. Soft black padding surrounds the sensor to prevent light leakage and maintain comfort of wearer as the sensor is held against the skin. This form factor alleviates issues with sensor application and contact with the skin. We custom fabricated the ear-seals to comfortably house the sensor such that it is held firmly against the wearer’s head behind the ear, in the specific location described above, in a self-contained unit housing the PPG sensor, battery, signal processing unit, and solid-state data storage. The SPYDR device in this study was installed within a military flyer’s helmet, such that it aligns with the helmet’s primary rotational axis and cranial support points, further reducing motion artifacts, but the sensor suite has potential for other applications as well.

The aim of this study was to determine if pulse rate (PR) and blood oxygen saturation (SpO_2_) could be accurately monitored using SPYDR installed in military flyer’s helmets. The gold standard arterial blood gas (ABG) PPG sensor validation analysis was conducted for SPYDR, along with two different commercially available finger sensors, to validate SPYDR for accuracy in measurements of PR and SpO_2_. In addition to accuracy against ABG, we also evaluated the SPYDR PPG sensor for continuity, and responsiveness for collecting data in hypoxic conditions in three simulated high-altitude, reduced oxygen environments to determine if it could produce reliable and accurate data for PR and SpO_2_ compared to two other commercially available PPG sensors at the finger and forehead. These tests conducted at varying oxygen levels, including moderate and severe hypoxic conditions, analogous to high altitude environments, were conducted using a reduced oxygen breathing device (ROBD). In this study, we determined we could accurately and reliably capture pulse rate and rapid changes in blood oxygen saturation behind the ear with SPYDR, with a faster response rate for changes in SpO_2_ compared to the PPG sensors at the finger and forehead.

## 2. Materials and Methods

### 2.1. Arterial Blood Gas Analysis

Arterial blood gas analyses was conducted at the Hypoxia Research Laboratory of the University of California San Francisco, an independent, university-based testing facility with no ties to pulse oximeter manufacturers. The lab specializes in evaluating the accuracy of pulse oximeters using an arterial blood gas analysis protocol as recommended by the United States Food and Drug Administration (FDA) and approved by the University of California San Francisco Committee on Human Research (approval number 10-00437).

This study included twelve (12) healthy, adult subjects (7 females, 5 males), ranging in age between 21 and 49 years of age. Skin tone varied from light to dark tone. Ethnicity varied to include 4 Asian, 3 Caucasian, 1 African American and 4 Multiethnic. All subjects were nonsmokers with no evidence of lung disease, obesity, or cardiovascular problems. For arterial blood collection for analysis, a radial arterial cannula was placed in the artery of the left or right wrist of the subjects. Each experiment consisted of two runs of five plateaus of oxyhemoglobin saturation between 70% and 100% for each the three sensors tested: #1 SPYDR (Figure 1), #2 Masimo Reference, and #3 Nellcor Reference. The level of oxyhemoglobin saturation was maintained at each plateau until the pulse oximeter readings stabilized and arterial blood samples were obtained. Two blood draws from an arterial cannula were taken sequentially during a stable plateau and analyzed. Each plateau was maintained for approximately 60 s with SpO_2_ fluctuating by less than 2–3%. The plateaus were nominally at 100% oxygen, room air saturation, 93%, 90%, 87%, 85%, 82%, 80%, 77%, 75%, and 70%. A total of 348 samples were obtained at the saturation plateaus across this span. Inspired O2 concentration was adjusted breath-by-breath using a computed saturation based on end-tidal PO2 and PCO2 as sampled by a mass spectrometer. SpO_2_ and pulse rate data were recorded every second using a laptop computer. Functional arterial SaO2 (HbO2/[Hb + HbO2]) was determined by multiwavelength oximetry using a Radiometer ABL-90 (Copenhagen, Denmark), which was calibrated according to manufacturer recommendations. Accuracy of SPYDR‘s pulse oximetry algorithms was determined in accordance with The U.S. Food and Drug Administration (FDA; Silver Spring, Maryland) parameters for manufacturers of pulse oximeters by comparing each SpO_2_ value with a “gold standard” SaO_2_ measurement collected by simultaneous co-oximetry of arterial blood samples.

### 2.2. Reduced Oxygen Breathing Device (ROBD) Testing

A total of five test subjects (four males, one female) were assigned to the test protocols. Of the test subjects, two were USN Corpsman, and three were Aircrew from VX-23. Test subjects were all briefed on the test objectives, test profile, as well as normal and emergency procedures. ROBD subject recruitment, experimentation, and data collection was performed independently by the United States Government. ROBD research subjects were identified and enrolled in accordance with Air Force Research Laboratory Institutional Review Board (IRB) Protocol FWR20170114H v3.00 “Multi-Channel Developmental Sensor Evaluation.” Approval date 7 May 2019. Each was exposed to a respiratory environment known to incur a physiological response consistent with hypoxic hypoxia while simultaneously monitored with three different sensors: 1) A commercial, FDA-approved finger sensor (Nonin Medical, Inc., Plymouth, MN, USA), 2) A commercial, FDA-approved forehead sensor (Masimo, Irvine, CA, USA), and 3) A HGU-68 flyer’s helmet (Gentex Corp, Carbondale, PA, USA) retrofitted with SPYDR earcups (Spotlight Labs, LLC, Sterling Heights, MI) (Figure 1). Subjects were seated in an F/A-18 ejection seat mockup and restrained by a five-point harness. Next, they were fitted with a standard-issue aviator’s oxygen mask (12/P or 20/P, Gentex Corp, Carbondale, PA, USA) connected to a reduced oxygen breathing device (ROBD, Environics, Toronto, ON, Canada). The ROBD is a previously verified compact, economical, closed-loop rebreather-type reduced-oxygen breathing device for hypoxia induction in humans. It simulates different altitudes by mixing a larger proportion of nitrogen into the breathing gas, thereby reducing the partial pressure of oxygen and causing a “normo-baric” hypoxic physiological reaction [37].

After checking all sensors for operability and conducting a mask leak-check, subjects were exposed to three profiles simulating different altitudes (Table 1). Each ROBD profile changed simulated “altitude” at a rate of 5000 feet per minute, and maintained each altitude for three minutes, allowing test subjects to reach a physiological equilibrium before moving to the next test step. ROBD Test#1 simulated an incremental altitude increase to 17,500 feet above Mean Sea Level (MSL), prior to an immediate return to “sea level,” coinciding with mask removal and breathing room oxygen. ROBD Test #2 featured the same incremental increase to a simulated altitude of 17,500 feet MSL, but then incrementally descended to “sea level” following the same intervals as the ascent. There was a 20-min break followings tests #1 and #2 whereby subjects could reacclimate to breathing room air at “sea level”. Higher altitudes corresponded to higher levels of nitrogen and lower levels of oxygen. The altitudes varied in plateaus by 5000 ft. intervals in order to allow subjects to acclimate at each level. The final ROBD profile included an incremental increase in simulated altitude to 25,000 feet MSL. Subjects remained at 25,000 feet until the observance of hypoxia symptoms as reported by the test subject or recorded by the observers as below 65% oxygen saturation, at which point they were immediately administered 100% oxygen.

Altitude decompression sickness (DCS) is a potential risk every time a pilot is in an unpressurized aircraft above 18,000 feet. However, there is very little evidence of altitude DCS occurring among healthy individuals at altitudes below 18,000 ft., which is why Test #1 and #2 were designed to remain below this altitude (17,500 ft.) and why test #3 was designed to simulate well above this altitude (25,000 ft) to ensure full high altitude exposure. Because of the physiological challenges and stress, and therefore also cognitive challenges and stress, cause by blood gases seeking equilibrium above this altitude, subjects were limited to going above 18,000 ft. to once per day, as per standard military flight procedures, as repetitive exposures to altitudes above 18,000 ft. within a short period of time without supplemental oxygen increases the risk of developing altitude DCS.

### 2.3. Statistical Analysis

Within thirty minutes of each test, raw data from SPYDR were compiled as a comma-separated variable worksheet (CSV) and emailed to the government representative and their designated representatives. Data obtained from the government’s laboratory instruments were similarly compiled and made available to Spotlight Labs. Within 24 h of test completion, a summary data analysis was emailed to the government comparing the performance of SPYDR to the commercially available, FDA-approved medical devices. The measurement of accuracy is presented as the Arms value and was calculated according to the latest draft of ISO 9919, Standard Specification for Pulse Oximeters. Arms Accuracy measurement was calculated as the square root of the sum of the bias squared and the standard deviation of the bias squared (√ (BIAS2 + SD2)). Typically referred to as the “RMS” figure of merit. This is the current method required by the FDA for the representation of pulse oximetry accuracy data. N (n) is the number of samples used to calculate the mean bias, standard deviation, and Arms value.

## 3. Results

### 3.1. Arterial Blood Gas Analysis Results

**Figure 2:**
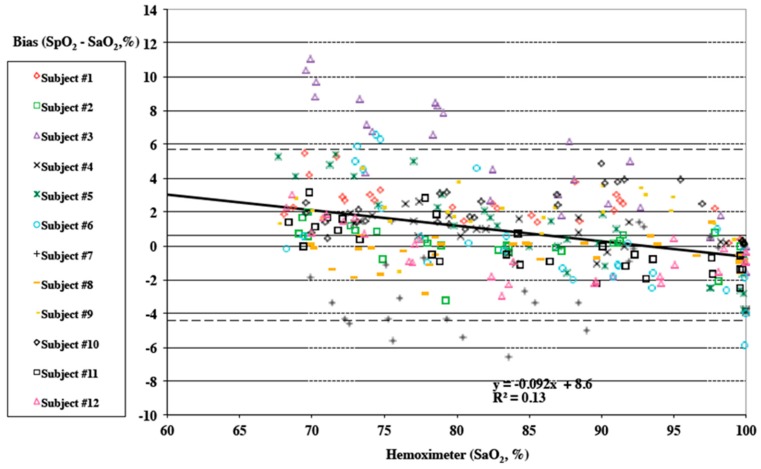
Bias plot of SpO_2_ determined by SPYDR vs. SaO_2_ determined by arterial blood gas analysis. A negative slop of the trendline suggests that, as commonly seen in reflectance oximetry, SpO_2_ values were more accurate at higher true values of SaO_2_ (e.g., clinically normal, 90–100%).

All data was independently analyzed and reported by the Hypoxia Research Laboratory at UCSF. Tables of mean, standard deviation, standard error, minimum, maximum, 95% confidence interval, count, and root mean square (RMS) are provided for each oximeter’s bias, and all oximeters combined in the following ranges of SaO2 (Hemoximeter): 60–80%, 80–100%, 60–100%, 70–100%, 50–60%, 60–70%, 70–80%, 80–90%, and 100%. In Table 2, mean, count, missing data, standard deviation, standard error, minimum, maximum, 95% confidence interval, count and root mean square are provided comparing SPYDR, Finger Sensor A, and Finger Sensor B bias. The FDA specifies an acceptable root mean square (RMS) error less than or equal to 3.5% for reflectance pulse oximeter accuracy testing across a 70% to 100% SaO_2_ range. As shown highlighted in Table 3, SPYDR had an RMS of 2.61%, well within the 70–100% SaO_2_ approved accuracy range. The mean bias for all subjects by SPYDR was 0.61%, suggesting overall accuracy was equally as accurate as sensor B (0.61% Mean Bias), but better than sensor A (0.66% Mean Bias). Figure 2, data is plotted as Hemoximeter data (SaO2) vs. pulse oximeter bias (SpO2–SaO2) as a Bland–Altman plot, according to their methods [38]. A different marker is used for each study subject. Linear regression is shown for all subjects combined, and the equation with R2 is shown on the plot. Mean bias is displayed as a solid horizontal line, and the upper and lower limits of agreement (mean bias ±1.96 SD*) are shown by dashed horizontal lines. A negative slop of the trendline suggests that, as commonly seen in reflectance oximetry, SpO_2_ values were more accurate at higher true values of SaO_2_ (e.g., clinically normal, 90–100%). Pulse oximeter data is taken as 5 s averages corresponding to the point of arterial blood analysis. Individual data points may be missed or excluded for dropped signals or failure of the oximeter signal to achieve an appropriate plateau. For the “pooled” plots, different markers are used for each pulse oximeter (Figure 2).

### 3.2. ROBD Results

The objective of ROBD testing was to determine how well SPYDR correlated to other commercially-available sensors commonly used for monitoring pulse oximetry and similar physiological metrics. To accomplish this, test data were recorded at 1Hz by SPYDR as well as forehead and finger PPG sensors. Data from a finger-mounted pulse oximeter was recorded every 5 s. To determine the statistical level of correlation between SPYDR and the other medical-grade sensors, data were analyzed using Pearson’s correlation coefficient. Covariance for a linear correlation were assessed for both SpO_2_ and Pulse Rate. Since the devices each captured data at different intervals, all data were first reduced to 5-s (0.2 Hz) sample rates. To account for beat-to-beat variability and device-specific signal processing differences, a six-measurement rolling average window was calculated (30 s window). Data were then plotted against each other, with SPYDR data on the *x*-axis and the control sensor on the *Y*-axis. Pulse rate data were similarly compiled and plotted.

**Figure 3:**
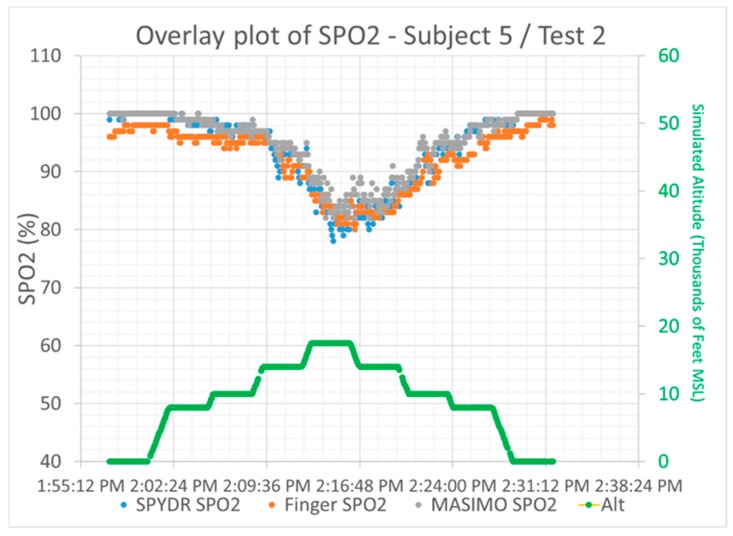
Exemplary overlay plot showing SpO_2_ output from SPYDR (blue), Nonin finger oximeter (orange), and Masimo forehead sensor (grey) show high degree of correlation between all sensors. Simulated altitude is shown as solid line at bottom (green), and all sensors performed as expected to reduced oxygen levels associated with changes in simulated altitude.

Throughout the duration of the ROBD test, SPYDR reported similar SpO_2_ measurements as the finger oximeter before the user reported observing their hypoxia symptoms, suggesting that it is an effective means of alerting aircrew to an impending hypoxic condition. Figure 3 is an exemplary overlay plot of SPYDR, finger, and forehead, as an example of what we saw of all sensors showing a high degree of correlation. Data from each sensor were first synchronized in time and plotted on the same chart to visually compare overall trends in SpO_2_ and PR. Correlation coefficients were determined by Pearson’s method, assessing covariance for a linear correlation. Data were then plotted with the SPYDR sensor on the *X*-axis and the control sensor (either the forehead or finger sensor) on the *Y*-axis. The solid line at the bottom of the plot indicates the simulated altitude with the ROBD running test profile #2. It is important to note that all three sensors tracked changes in SpO_2_ as expected, with decreasing values as altitude increases, as higher altitudes were simulated with lower oxygen levels.

**Figure 4:**
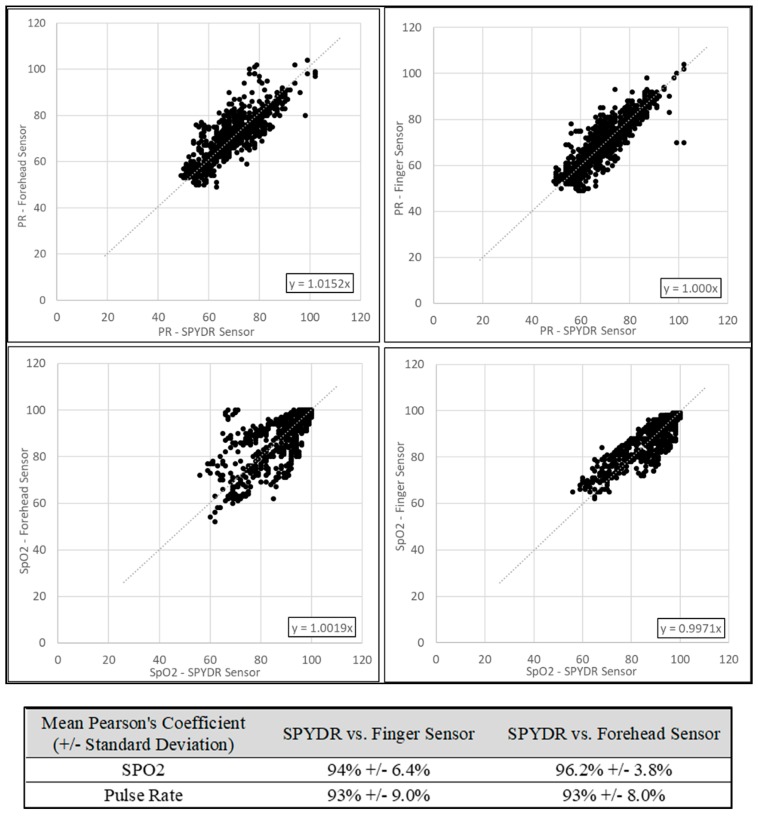
Correlation plots for (**A**) forehead pulse rate vs. SPYDR pulse rate; (**B**) finger pulse rate vs. SPYDR pulse rate; (**C**) forehead SpO_2_ vs. SPYDR SpO_2_; and (**D**) finger SpO_2_ vs. SPYDR SpO_2_. The linear relationship shown in each case suggests a high correlation between SPYDR and the respective commercial sensor.

Figure 4 represents the correlation plot of SpO_2_ and PR for the SPYDR sensor versus the forehead and finger sensors. Data shown are compiled from all test subjects (n = 1070). Compared to the commercial forehead sensor, SPYDR had a Pearson’s coefficient of 96% for PR, and 92% for SpO_2_, suggesting that SPYR’s measurements were as accurate as both commercial sensors, showing a strong correlation. Correlation of SPYDR to the commercial finger sensor was 94.0% for PR, and 96.1% for SpO_2_.

**Figure 5:**
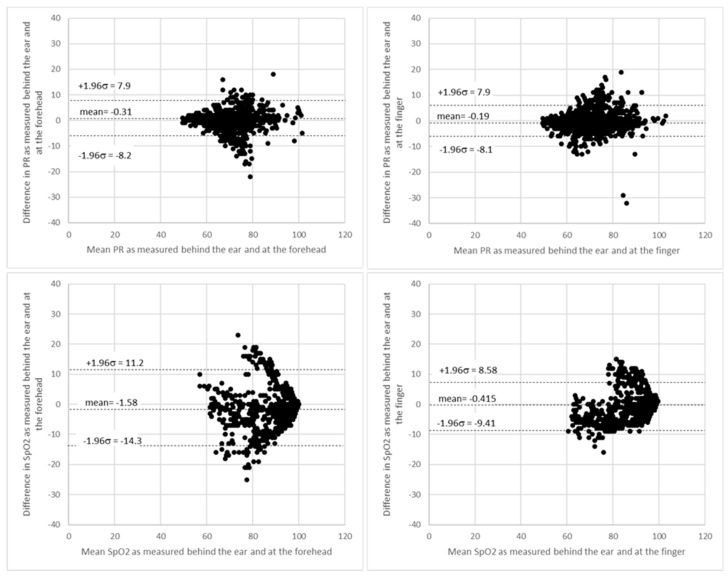
Bland–Altman plots of PR and SpO_2_ measurements comparing SPYDR with finger and forehead PPG. The x and y axes indicate the mean and difference of the photoplethysmography (PPG)-derived and the corresponding reference respiratory frequencies, respectively. The dotted lines show the mean bias. The limits of agreement (LoA) mean ±1.96 SD interval of difference are given with the solid lines.

**Figure 6:**
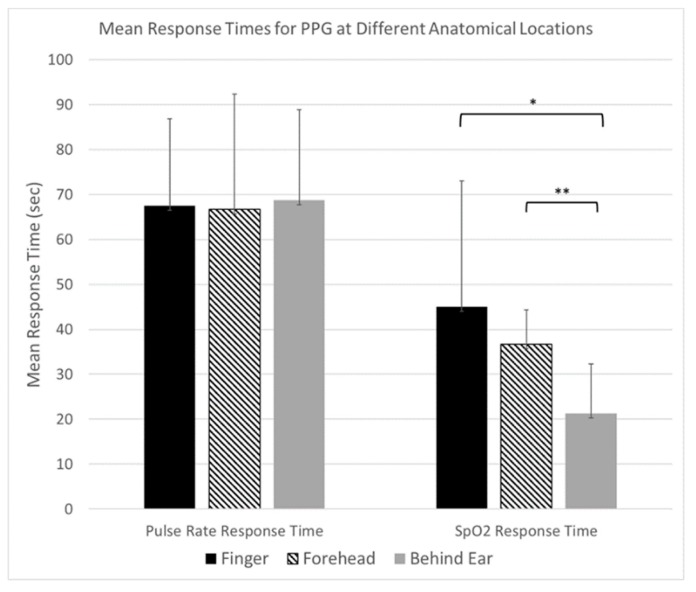
Mean response times comparison for all three locations of PPG readings upon administration of 100% oxygen following hypoxic conditions: Finger (black), Forehead (striped), and Behind the Ear (gray), for Pulse rate and SpO_2_ readings, showing similar response times for Pulse Rate and a significantly faster response time for SpO_2_ changes behind the ear location compared to both finger and forehead. * - *p* = 0.077 by students t-test, two-tailed, paired sample, n = 4 ** - *p* = 0.19 by students t-test, two tailed, paired, n = 3.

**Figure 7:**
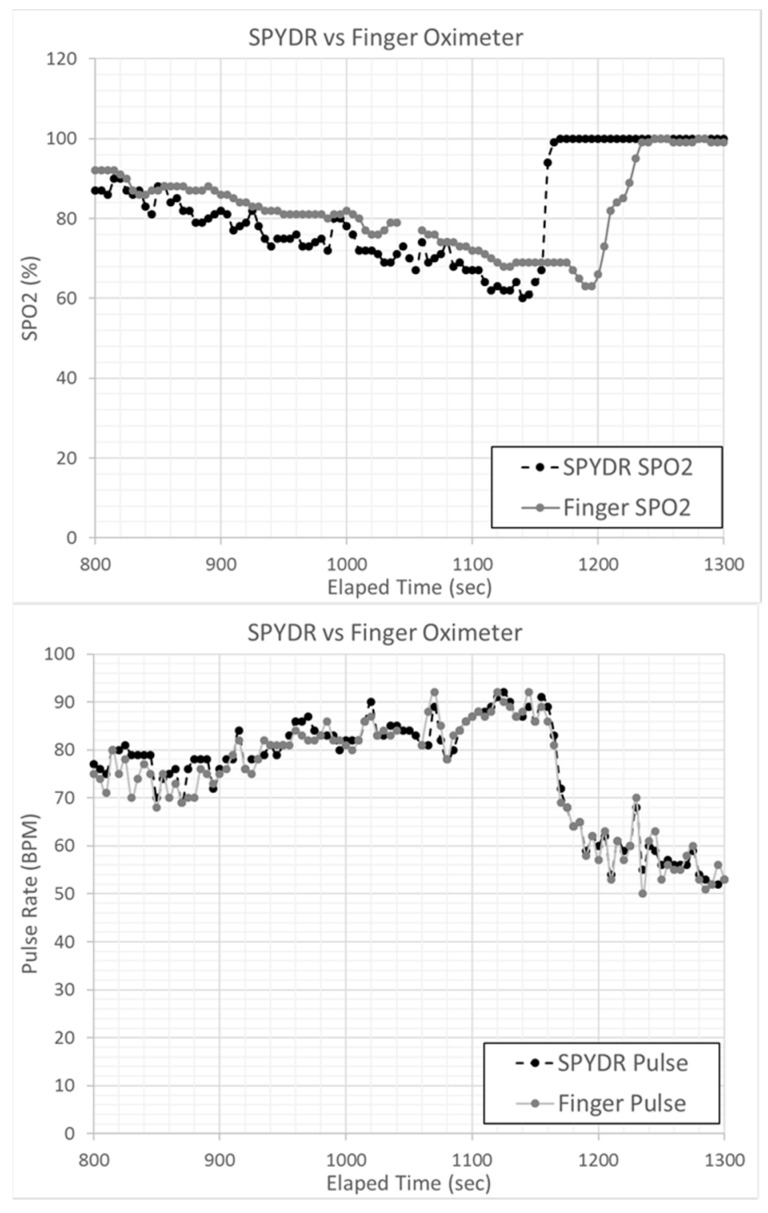
Exemplary overlay plot of SpO2 levels for SPYDR and a finger-based pulse oximeter. In (**a**) after initiating 100% oxygen at the lowest SPO2, SPYDR detected normal SpO2 levels 50 s faster than the finger-based sensor. (**b**) pulse rate comparison between SPYDR and finger for the same time period as SPO2 readings, validating the time synchronicity of the devices.

Figure 5 shows Bland–Altman plots of PR and SpO_2_ measurements comparing SPYDR with PPG readings at the finger and forehead. The x and y axes indicate the mean and difference of the PPG-derived and the corresponding reference respiratory frequencies, respectively. The dotted lines show the mean bias. The limits of agreement (LoA) mean ±1.96 SD interval of difference are given with the solid lines. ROBD test profile #3 simulated altitudes up to 25,000 ft. Following exposure to these hypoxic conditions, subjects were administered 100% oxygen. Importantly, the study shows a faster response time as seen by SPYDR to changes in SpO_2_, upon the administration of 100% oxygen following hypoxic conditions in the ROBD studies compared to the finger sensor in Figure 6 and Figure 7, while simultaneously closely recording the same values for pulse rate.

The response rate to slowly decreasing blood oxygen levels appeared similar for all three sensors. During the entirety of the test, a noticeable decline in SpO_2_ levels measured by all sensors was observed prior to hypoxia symptoms being reported by the user. However, after 100% oxygen was administered to test subjects experiencing severe hypoxia (Table 1. Profile #3), SpO_2_ indications also returned to normal (>95% SpO_2_) as much as 50 s faster for SPYDR than peripheral sensors (Figure 6 and Figure 7) because oxygenated blood reaches the brain via the carotid artery (and thus, the SPYDR sensor) faster than the capillaries of the finger, especially in conditions of prolonged or severe hypoxia.

**Figure 8:**
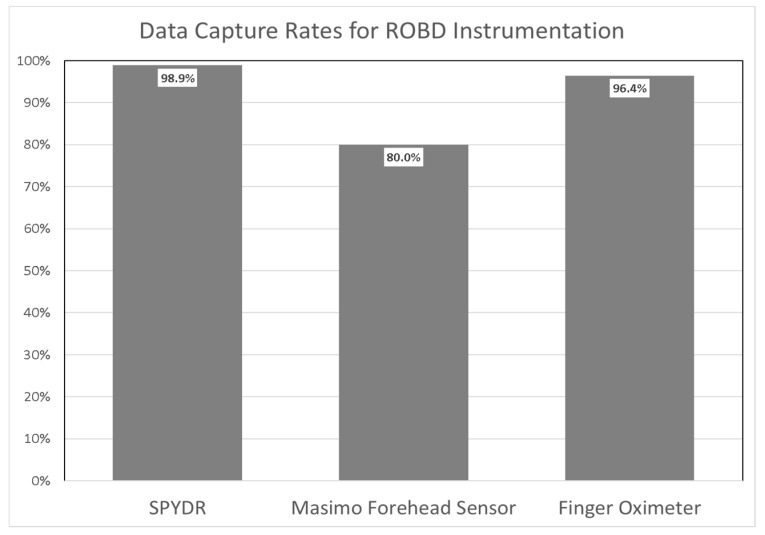
Data capture rates for SPYDR, Forehead, and Finger sensors used to monitor pulse rate and blood oxygenation (SPO2) during ROBD verification and validation. SPYDR capture rate was 98.9%, Forehead sensor was 80.0%, and Finger sensor was 96.4%.

Data dropouts due to motion or low perfusion by SPYDR were uncommon. Throughout 15 trials, SPYDR’s mean data capture rate was 98.9%, and the lowest data capture rate was 92%. Data capture rates were consistently above 95% for all test subjects, with 100% capture rates for all aircrew tested. Data dropouts for the peripheral sensors were more common. The finger sensor only captured 96.4% of data, and the forehead sensor only captured 80% of SpO_2_ and PR data for the same time period (Figure 8). The high capture rate of SPYDR is due largely to having two earcups installed, providing redundancy if needed in a challenging environment. In some cases, one ear cup will stop tracking for short periods during the flight, and the other earcup will essentially” fill-in” the gaps of missing data.

## 4. Discussion

Previous research has discovered a number of factors that typically affect the accuracy of PPG readings in general, including the anatomical location of measurement, implementation of skin contact, application force, motion artifact, ambient temperature, and anatomy of the subject [3,4,15,39]. The objective of this research to meet these challenges by validating the accuracy and reliability of a novel device SPYDR, a PPG sensor suite, and its location behind the ear for measuring oxygen saturation (SpO_2_) and pulse rate (PR), by comparing the device’s responsiveness to two commonly-used, commercially available, FDA approved PPG sensors at the finger and forehead.

We believe a number of benefits of SPYDR likely contribute to its accurate and reliable readings. This behind the ear location is optimal because of lack of hair follicles in the area, in both men and women, as opposed to an area in front of the ear where the readings may be encumbered by facial hair or a low hairline. Also, the behind the ear location is well suited for accuracy and reliability of readings because of low musculature in the area precluding excess movement, there are no major veins or arteries, and low movement of the cranium as opposed to the appendages, as the head typically moves less than the extremities. Motion artifacts, one of the largest limiting factors in PPG measurement accuracy, are usually caused by movement of the PPG sensor over tissue, skin deformation, or blood flow [35,40]. With SPYDR, we overcame many of the limitations of motion artifacts by location and design for the application of the sensor to the skin with the ear-seal design, which additionally blocks out disruptive ambient light.

As a first step in evaluating and validating the accuracy of the device, SPYDR was tested against simultaneous arterial blood gas (ABG) readings, the gold standard for novel PPG sensor validation. ABG readings were conducted by the highly renowned independent, university-based testing facility, the Hypoxia Research Laboratory of the University of California San Francisco, with testing protocols designed to generate data suitable for submission to the US Food and Drug Administration needed for novel PPG device approval. In these tests, continuous airway gas analysis by mass spectrometer is utilized, and pulse oximeter readings with SPYDR were compared to the direct arterial blood gas levels with multiwavelength oximetry. The root mean square (RMS) value takes into account both mean and standard deviation on the calculation, with a value of <3.5% considered accurate for reflectance PPG sensors. As shown in the results, SPYDR meets the standard criterion for PPG sensor accuracy with an RMS value of 2.61 for a hemioximeter range of 70–100%, as seen in Table 2 and Table 3.

ROBD tests were conducted to further explore the potential and validate the accuracy of SPYDR by comparing it to PPG sensor readings at the finger and forehead under conditions that cause dynamic changes to blood oxygen levels. In this test, SPYDR detected changes in oxygen saturation levels, while closely tracking a consistent pulse rate in hypoxic conditions faster than both sensors, and up to 50 s faster than the finger location (Figure 6 and Figure 7), as would be expected due to its location at the extremities. We also saw a general trend of a faster response time to rapid changes in SpO_2_ levels by SPYDR compared to the forehead sensor, although the standard deviation was too high to consider the differences to be statistically significant. However, we believe the inability to detect a statistically significant faster response of SPYDR over the forehead location is due to the small test subject size, as in our own unpublished studies and observations we consistently find SPYDR to have the fastest response time behind the ear compared to any other site.

These factors combined suggest that monitoring pulse rate and arterial oxygen saturation with PPG behind the ear might circumvent the numerous effects of peripheral vasoconstriction and decrease the time to detect a hypoxic event. We were able to successfully validate the accurate performance of SPYDR in collecting pulse oximetry data at hypoxic levels of SpO_2_, while also monitoring the speed at which the device is able to detect a return to 100% SpO_2_ levels upon administration of 100% oxygen to subjects following hypoxic symptoms. Test subjects reported high satisfaction with SPYDR, reporting zero comfort or interference issues. As observed in previous tests, SPYDR earcups left uniform imprints on the user’s heads around the ears, allowing for identification of sensor placement after flight, but which did not cause pain of discomfort to the subjects.

Human cognition and survival are critically dependent on the availability of oxygen to the brain. However, the brain has no oxygen storage capacity of its own and therefore requires a continuous supply. Lack of brain tissue oxygenation can lead to a loss of nerve cell function in a matter of seconds. Previous studies have shown that a decline in core arterial oxygen saturation is delayed if the measurement is performed peripherally compared to a central location [1,6,7,8,9]. A key feature of SPYDR’s behind the ear location is its closer proximity to the brain in line directly from the heart, as opposed to an extremity-mounted sensor, creating a more relevant analogue for cerebral oxygenation. It is ideal for determining levels of oxygen going directly to the brain, as oxygenated blood reaches the brain (via the carotid artery) faster than the extremities (capillaries of the finger) [41]. The value of this location was evidenced by SPYDR’s ability to detect changing SpO_2_ measurements prior to the observance of hypoxia symptoms reported in the subject, significantly faster than the other sensors at finger and forehead. Based on these anatomical and physiological advantages, we predict faster measurements with SPYDR at the onset of hypoxic conditions compared to sensors at other sites as well. We also believe our location is advantageous to other locations on the ear, such as in the ear-canal and earlobe because the ear-canal is highly subjected to motion artifacts from chewing and speaking, and the earlobe is consider an extremity that is susceptible to inaccuracies when peripheral perfusion is compromised, performing with less accuracy than a finger sensor in hypoxic conditions, as shown by Ross et al. [23,28,42] 

The faster detection rate of SPYDR to changes in blood oxygen saturation, measuring behind the ear, as compared to the finger or forehead, could provide earliest detection of hypoxic episodes. The development of alerting algorithms based on these data streams could likely provide users with key information and pre-emptive warning of impending emergencies, which have historically led to loss of life and destruction of property. This early detection capability could then be connected to an early warning signal, as is an ultimate goal of this device. Since these hypoxia experiments were performed on healthy volunteers, we are so far not able to make conclusions on the clinical usefulness of this technique. However, this was also outside the scope of the current study. Before a possible introduction in clinical practice, further investigation of the technique under more dynamic conditions should be considered.

As the aviation community attempts to maximize training effectiveness and human performance while mitigating risk, an objective tool to quantify cognitive status and draw attention to degraded cognitive performance is needed. To date there are no reliable devices within tactical aviation to monitor oxygen levels of fighter pilots. This is critical because it is hypoxia and loss of consciousness of the pilot, not equipment failure, that leads to most fatal crashes. Under periods of high acceleration, the body’s cardiac output is placed under considerable strain. The heart’s efforts to circulate oxygen rich blood to the brain are countered by the aircraft’s centripetal acceleration, causing reduced oxygenation of the brain and, in severe cases, an ischemic loss of consciousness. SPYDR represents an important opportunity to collect data for a relative performance index and could ultimately improve current risk mitigation techniques, as well as better detect and alert pilots to a degraded cognitive status due to hypoxia. Ease of application and implementation extend the quantity of applications and industries for SPYDR. Future studies exploring its accuracy in highly dynamic environments associated with tactical flight would provide further valuable information for validating the feasibility of SPYDR in the field. Additionally, with continual advances in data science and machine learning, expanded research on in-flight data derived from SPYDR may result in enhanced biometric quantification of human health, performance, and cognitive status in addition to early warning signals for hypoxia.

The standard FDA testing protocol for novel PPG sensor suites requires that all subjects be healthy and free of preexisting conditions, defined primarily as being nonsmokers with no evidence of lung disease, obesity, or cardiovascular problems, that would negatively impact arterial blood gas results and put the subjects at risk. Therefore, one limitation of this study is that all subjects were classified as “healthy” and the results here do not show if there will be similar readings in subjects with preexisting health problems, such as lung disease or cardiovascular illness. Also, while some applications within this study are not those typically seen in patients, such as simulated high altitudes, the hypoxic conditions of reduced blood oxygen tested under these criteria can be analogous to physiological responses seen in other pathological or acute adverse conditions. We therefore would expect the SPYDR sensor suite at this specific behind the ear location to still perform reliably in patients with preexisting health conditions in future studies, creating great potential for this technique in the medical field as well.

The data presented in this preliminary study suggest that the SPYDR sensor suite reading behind the ear provides accurate measurements for physiological parameters of cardiovascular performance that is as accurate and reliable as finger and forehead PPG sensors with a faster response to detecting changes in blood oxygen saturation levels.

## Figures and Tables

**Table 1:** Outline of reduced oxygen breathing device (ROBD) test altitudes. Each simulated altitude was maintained for 3 min, and all altitude changes were accomplished at a rate of 5000 feet per minute. There was a 20-min break between test #1 and 2 whereby subjects were breathing room air. The final descent “sea level” coincides with room air, while 100% oxygen was administered in test #3 following hypoxic conditions.

Test #1	Test #2	Test #3
8000 MSL	8000 MSL	8000 MSL
10,000 MSL	10,000 MSL	10,000 MSL
14,000 MSL	14,000 MSL	14,000 MSL
17,500 MSL	17,500 MSL	17,500 MSL
Sea Level	14,000 MSL	21,000 MSL
	10,000 MSL	25,000 MSL
	8000 MSL	100% Oxygen
	Sea Level	

**Table 2:** Bias for SPYDR (reflectance oximetry) vs. two commercial, FDA-approved finger sensors (Sensor A and B) for 70–100% SaO_2_. All data were independently analyzed and reported by the Hypoxia Research Laboratory at UCSF.

	SPYDR	Finger Sensor A	Finger Sensor B
Mean	0.61	0.66	0.61
Count	314	308	308
Missing Data	8	14	14
Standard Deviation	2.54	1.6	1.28
Standard Error	0.14	0.09	0.07
95% Confidence Interval	0.28	0.18	0.14
Limits of Agreement	−4.44 to 5.67	−2.52 to 3.83	−1.93 to 3.14
Maximum	9.7	5.8	4.5
Minimum	−6.6	−4.7	−4.2
Root Mean Square	2.61	1.72	1.41

**Table 3:** Bias for SPYDR broken down by decadal ranges from 70–100% SaO_2_. At all ranges, SPYDR exceeded FDA criterion for accuracy of reflectance pulse oximetry (<3.5% root mean square (RMS)), highlighted in grey. Data as presented were independently analyzed and reported by the Hypoxia Research Laboratory at University of California San Francisco.

SPYDR Bias				
Hemoximeter Range	70–80%	80–90%	90–100%	70–100%
Mean	1.64	0.37	−0.12	0.61
Count	109	79	126	314
Missing Data	0	1	7	8
Standard Deviation	3.02	2.25	1.9	2.54
Standard Error	0.29	0.25	0.17	0.14
95% Confidence Interval	0.57	0.5	0.34	0.28
Limits of Agreement	−4.47 to 7.74	−4.16 to 4.90	−3.91 to 3.67	−4.44 to 5.67
Maximum	9.7	6.2	5	9.7
Minimum	−5.6	−6.6	−5.9	−6.6
Root Mean Square	3.42	2.26	1.9	2.61
